# Timing of imagery rescripting during schema therapy for borderline personality disorder: the LUCY trial

**DOI:** 10.3389/fpsyt.2023.1204439

**Published:** 2023-12-13

**Authors:** Annemieke Koppeschaar, Nathan Bachrach, Arnoud Arntz

**Affiliations:** ^1^Parnassia Groep Academy, The Hague, Netherlands; ^2^Academic Centre for Trauma and Personality, Amsterdam, Netherlands; ^3^Department of Medical and Clinical Psychology, Tilburg University, Tilburg, Netherlands; ^4^Department of Personality Disorders, GGZ-Oost Brabant, Helmond, Netherlands; ^5^Department of Clinical Psychology, University of Amsterdam, Amsterdam, Netherlands

**Keywords:** borderline personality disorder, schema therapy, imagery rescripting, treatment, randomized controlled trial

## Abstract

**Background:**

Early childhood adversity plays an important role in the etiology of borderline personality disorder (BPD). Current evidence suggests that trauma treatment for patients with BPD can be performed safely and that early trauma treatment has a positive effect on the course of PD. However, there is a scarcity of RCTs comparing the effects of the timing of trauma treatment during schema therapy (ST) for BPD on BPD severity. Therefore, the LUCY trial investigates the effects of the timing of trauma treatment by comparing early trauma treatment using imagery rescripting (ImRs) on the course of BPD during ST to trauma treatment in the middle of the treatment course.

**Methods:**

In this multicenter RCT, two conditions are compared among 73 individuals with BPD. The participants receive combined individual and group ST in both conditions. However, in condition (A), participants directly start ImRs in the individual sessions in months 2–4, and in condition (B), participants receive ST-as-Usual (STAU), in which ImRs is not allowed during months 2–4. The treatment follows ST treatment protocols, consists of a fixed combination of individual sessions and group sessions with a maximum of nine patients, and has a maximum duration of 25 months. The primary outcome is change in BPD severity, which is assessed using the Borderline Personality Disorder Severity Index-5 by independent raters blinded to the treatment. Secondary outcome measures include treatment retention, disconnection/rejection schemas, general functioning, posttraumatic stress disorder symptoms, general psychopathological complaints, quality of life, happiness, schemas, and schema modes. Multilevel analysis will be performed to analyze and compare changes in BPD severity between conditions and generalized linear mixed model analyses to test predictors and moderators.

**Discussion:**

This study will increase the knowledge on whether trauma treatment early in therapy positively affects the course of BPD manifestations during ST. When the early application of ImRs leads to a faster decrease in BPD manifestations, the treatment of BPD patients might be shortened, leading to improved treatment outcomes and decreased healthcare expenses. Moreover, the planned sub-studies will expand our knowledge of how ST works and the factors that influence the outcome of treatment.

## Introduction

This article describes the study protocol of the LUCY trial (Legitimizing the Use of Childhood memory rescripting early in the first Year), a multicenter randomized controlled trial (RCT) on the effects of the timing of trauma treatment by imagery rescripting (ImRs) on the course of borderline personality disorder (BPD) manifestations during schema therapy (ST). Among the major evidence-based treatments for BPD, ST has a unique standpoint toward adverse childhood experiences of BPD patients, compared to other evidence-based treatments for BPD [e.g., dialectic behavioral therapy (DBT), mentalization-based treatment (MBT), and transference focused psychotherapy (TFP)], because in comparison with other evidence-based treatments for BPD, traditionally in ST, adverse childhood events are always addressed and processed during ST with ImRs. ImRs is an ST technique that is a highly effective evidence-based stand-alone trauma processing technique for post-traumatic stress disorder (PTSD) and a range of other disorders (1–5). Only more recently, specific trauma-focused DBT and MBT programs have been developed for BPD patients with comorbid PTSD, underscoring the importance of trauma processing in the treatment of patients with PTSD and BPD (6–8). In ST, ImRs is used to process both traumatic experiences that contributed to the development of maladaptive schemas but did not lead to a comorbid PTSD, such as experiences of neglect and emotional abuse, as well as in the case of adverse experiences that (potentially) lead to comorbid PTSD such as abuse and violence experiences (4, 5, 9). Because BPD is related to various types of childhood adversities, it is important to process important experiences that contribute to the maintenance of BPD symptoms during treatment (10).

Trauma processing in the treatment of BPD patients appears important for several reasons. Experiences of early adverse childhood experiences are assumed to play a significant role in the etiology of BPD (11). On average, individuals with BPD have a 13.9 times higher chance of experiencing childhood adversity than non-clinical individuals (12). Compared with other psychiatric groups, BPD patients have a 3.15 times higher chance of experiencing childhood adverse events (13), and they often report a wide range of negative developmental experiences and frequently experience multiple childhood adverse events such as abuse and neglect (10, 12, 14). In addition, adverse events in (early) childhood contribute to the development of early maladaptive schemas (basic mental representations of the self, relationships with others, and the world) and dysfunctional schema modes (encompassing the current emotional, cognitive, and behavioral states of an individual) (15). These schemas have been found to mediate the link between early life experiences and the emergence of personality disorders (16). Finally, the prevalence of PTSD among BPD patients is relatively high, ranging from 34 to 46% (17). This specific group of BPD patients with comorbid PTSD is characterized by a higher level of exposure to traumatic events in interpersonal relationships during both childhood and adulthood compared to BPD patients and PTSD patients (13).

There is evidence that trauma-focused therapy for PTSD can be safely performed in patients with BPD (17). In addition, several studies have investigated the results of combined PD and PTSD treatment on co-occurring personality disorder traits [e.g., (6, 7)]. These studies found a relationship that combined treatment is feasible, acceptable, and safe to administer and may lead to larger improvements in PTSD and BPD than a PD treatment alone. However, little is known about the effects of targeting memories of adverse (childhood) events early in treatment in people with a personality disorder (PD) who do not have comorbid PTSD. A recent RCT by Hafkemeijer et al. (18) compared five sessions of eye movement desensitization and reprocessing (EMDR) therapy (5 weekly sessions of 90 min, 7) to a waiting list control condition (a period of 5 weeks). After 5 weeks of EMDR or 5 weeks of the waiting list control condition, patients in both conditions received ST-as-Usual (STAU) for their PD. After 3 months, significant improvements with medium effect sizes were found for psychological symptoms, psychological distress, and personality functioning in the EMDR condition compared to the STAU condition, indicating that early trauma treatment can be beneficial in the treatment of individuals with PDs (18).

Contrary to these recent positive findings of early trauma treatment, current ST protocols for BPD prescribe trauma treatment in the middle of the treatment course (9). To date, an important reason for postponing trauma processing to the middle phase of ST is the idea that the therapeutic relationship is not yet strong enough, and the patient is not yet ready (9). Trauma processing with ImRs forms an integral and important part of ST for BPD but is usually postponed to the mid-phase of therapy (9), which might be suboptimal given recent positive findings with regard to the effects of early trauma treatment on the course of PD's (18). Trauma processing earlier in therapy might improve the effectiveness of ST, which might lead to shortening the treatment duration and thereby an increase in cost-effectiveness. Therefore, the LUCY trial investigates the effect of early trauma treatment by ImRs on the course of BPD during ST, compared to trauma treatment in the middle of the treatment course.

## This study

Initial evidence suggests that trauma treatment for PTSD can be performed safely in BPD patients, and no stabilization is needed (17). Nevertheless, randomized studies comparing the effects of trauma processing early in ST on BPD severity compared to treatment with no or later trauma processing are lacking. Therefore, this study investigates the effects of early trauma treatment with ImRs on BPD severity during ST.

The central goal of the LUCY trial is to investigate whether ImRs applied early in therapy positively impacts the course of BPD manifestations in ST. In addition, the effects of ImRs on a set of secondary outcomes will be explored, including general functioning, PTSD symptoms, general psychopathology, quality of life, happiness, schemas, and schema modes. A multicenter randomized trial is performed in regular mental healthcare settings. Participants with a primary diagnosis of BPD receive a combination of individual and group ST. Two treatment arms are compared. Both arms start with 1 month of getting acquainted with the ST concepts, forming a working relationship, and formulating a case conceptualization. Thereafter, in condition (A) early ImRs, patients start with ImRs in months 2–4 in their individual sessions, and in condition (B) STAU, patients receive schema therapy as usual (no ImRs allowed during months 2–4). We hypothesize, in accordance with recent findings that show positive effects of early trauma treatment on the course of PD, that recovery from BPD (i.e., reduction of BPD severity) benefits from ImRs applied early in therapy (18).

Second, attrition is quite high in BPD treatment [on average around 40% after one year of treatment in a meta-analysis by Arntz et al. (19)]; if it is indeed the case that patients are not ready for early ImRs, then dropout could be even higher in the early ImRs condition (9). It could however also be the case that providing early trauma processing with ImRs could reduce dropout because trauma-related symptoms, which are associated with high burden and high dropout rates, are treated effectively early. A second objective of this RCT is, therefore, to investigate whether or not trauma processing early in treatment leads to increased dropout from treatment.

Third, the effect of early application of ImRs on the working relationship with the therapist, reported by the patient, is investigated. Early application of ImRs is expected to positively affect the therapeutic relationship in the individual ST compared to usual ST, because in the ImRs, the condition therapist potentially meets the needs of the patients sooner as compared to STAU in which ST techniques can be used that potentially less directly meet the needs of the patient, such as cognitive and behavioral techniques. In ImRs, imagery is used to modify the meaning of traumatic memories by the patient imagining a new script that meets the needs of the patient, thereby directly meeting the unmet need of the patient. It is hypothesized that this could possibly lead to a more secure attachment and improved trust, in other words, a better working alliance. An indication of this comes from a recent qualitative study, in which patients indicated that the therapist being both confrontational (and not avoidant) and able to meet the patient's needs during ImRs was helpful to improve the working alliance (20).

Fourth, the anticipated outcome is that early implementation of ImRs will result in a quicker reduction of the levels of the disconnection and rejection domain schemas, including abandonment/instability, mistrust/abuse, emotional deprivation, defectiveness/shame, and social isolation/alienation, as evaluated by the Young Schema Questionnaire (YSQ), than in usual ST, due to earlier meeting of the needs of the patient.

In summary, multiple explanations are possible for a (possible) positive effect of early ImRs on BPD severity. Therefore, in a sub-study, several mechanisms of change of reduction of BPD severity will be investigated; more specifically, mediation of reduction of BPD severity by schemas, schema modes, working alliance, and PTSD symptoms will be investigated. In addition, in a second sub-study, a qualitative study will be performed in which the experience of therapists and patients with early ImRs will be investigated with semi-structured interviews to gather knowledge on the experiences about the application of early ImRs which will also be used to optimize the application of ImRs early in ST. Finally, the effects of differences in ST dosage (outpatient vs. intensive day clinical treatment—provided by one of the sites, see treatment paragraph for details about the dosage differences) on the effectiveness of ST will be explored.

## Method

### Design

This study design is a multicenter RCT, with two arms (*n* ≥ 73). Both treatments consist of a combination of individual and group sessions. There are two versions of individual ST, to which participants are randomly assigned: (A) experimental ST, in which ImRs is delivered in every individual session during months 2–4 (early ImRs), and (B) STAU, in which patients receive ST-as-Usual, that is, ImRs is not allowed during months 2–4. Therapists in this condition are allowed to use other ST (experiential) techniques, such as chair work and cognitive and behavioral techniques, but are not allowed to use ImRs. After 4 months, therapists are free to use all ST techniques that they think are appropriate and helpful. This study is being carried out in five corporate mental healthcare centers in the Netherlands: GGZ Oost Brabant (locations Helmond and Oss), and PsyQ locations Amsterdam, Rotterdam, and Zaandam. During the study, the PsyQ location in Amsterdam was taken over by the Academic Center for Trauma and Personality (ACTP).

In two PsyQ sites (Amsterdam and Rotterdam), several participants also took part in the Borderline Optimal Treatment Selection (BOOTS) study. The BOOTS study compares the effectiveness of DBT to ST and investigates the optimal treatment selection among individuals with BPD (21). Eighteen participants participated in the BOOTS trial and were randomized to the ST arm. They completed the assessments needed for the BOOTS and the LUCY trial, after which they were randomized to one of the two arms of the LUCY trial.

The study protocol was approved by the ethical committee of the University of Amsterdam (2019-CP-10845). This study is registered at the International Clinical Trial Registry Platform, registration number NL NL7965, first registered on 15 August 2019 and fulfills the World Health Organization Trial Registration Data Set. The procedures adhered to applicable guidelines and regulations. Any changes in the protocol will undergo formal amendments. The ethical committee of the University of Amsterdam will review the amendments, and, upon approval, they will be included in the trial registration.

### Time frame

The trial was registered on 15 August 2019, and the first eligible patient started treatment on 31 October 2019. The last patient started treatment on 24 January 2023 and is expected to complete the treatment on 24 July 24. The last assessment is planned to take place on 24 January 2026, and data collection will be completed after that assessment.

### Patients

Patients were included in the study if they (2) had a primary diagnosis of BPD [assessed with the Structured Clinical Interview for Personality Disorders (SCID-5-PD)] (22), (2) had a score of 20 or above on the Borderline Personality Disorder Severity Index [BPDSI, (23)], (3) were between 18 and 65 years old, and (4) were able to understand, read, write, and speak Dutch (or English for the Amsterdam site, where separate Dutch and English ST is offered). Patients were excluded if they had (1) an alcohol or drug dependency and were in need of a clinical detox (after 3 months of abstinence participation is possible), (2) a comorbid psychotic disorder (when > 1 year in full remission inclusion is possible), (3) an antisocial PD with a history of physical interpersonal violence in the last 2 years, (4) DSM-5 bipolar disorder, type 1 (current or past; if there has been no manic episode in the last year, patients are included) (24), (5) an acute suicide risk, (6) an IQ < 80, (7) received ST of any kind (e.g., individual, group, inpatient, outpatient, and day treatment) in the past year, and (8) were not able to commit to group therapy sessions of 90 min and individual sessions of 45–60 min once a week for 2 years within the treatment period. Patients were asked not to start any other psychological treatment or medicine during screening or during the ST treatment or waitlist period, and prior to beginning ST treatment, medication levels had to remain constant for a minimum of 3 months if not stopped. Non-PD-focused supportive treatment may be continued during the waitlist period and during screening but not during ST treatment in the study and 1-year follow-up period.

### Sample size

At least *N* = 73 participants were planned to be included. Using the approximation proposed by Twisk (25), and assuming an average correlation of 0.5 between measurements [as in (26)], this suffices, given the seven repeated measurements, to reach 80% power to detect a medium effect size of *d* = 0.5 between the treatment conditions. Using the more sophisticated power analysis approach proposed by Moerbeek et al. [(27), p. 191] for multilevel tests of a difference in linear slopes assessed with multiple (i.e., 7) measurements, for a medium effect size (*d* = 0.5) tested at a two-tailed significance level of 0.05, *N* = 39 suffices to reach 80% power. Starting with *N* = 73 and taking into account an early study dropout of 10%, the resulting N=66 suffices to reach > 95% power to detect a medium effect size (*d* = 0.5) and 80% power to detect a small-to-medium effect size of *d* = 0.38. An effect size of *d* = 0.5 between slopes is equivalent to a mean difference between conditions of approximately five points on the BPDSI at the last assessment, which is a relevant difference. Moreover, the sample size of N = 73 suffices to reach more than 80% power to detect at a significance level of 0.05, a difference of 30% in dropout rates during the first year between 20% [which is the approximate dropout rate of ST in individual-group format at 1 year; (26)] and 50%, which would be a substantial and alarming increase in the experimental treatment (25, 28).

### Recruitment

**Figure 1:**
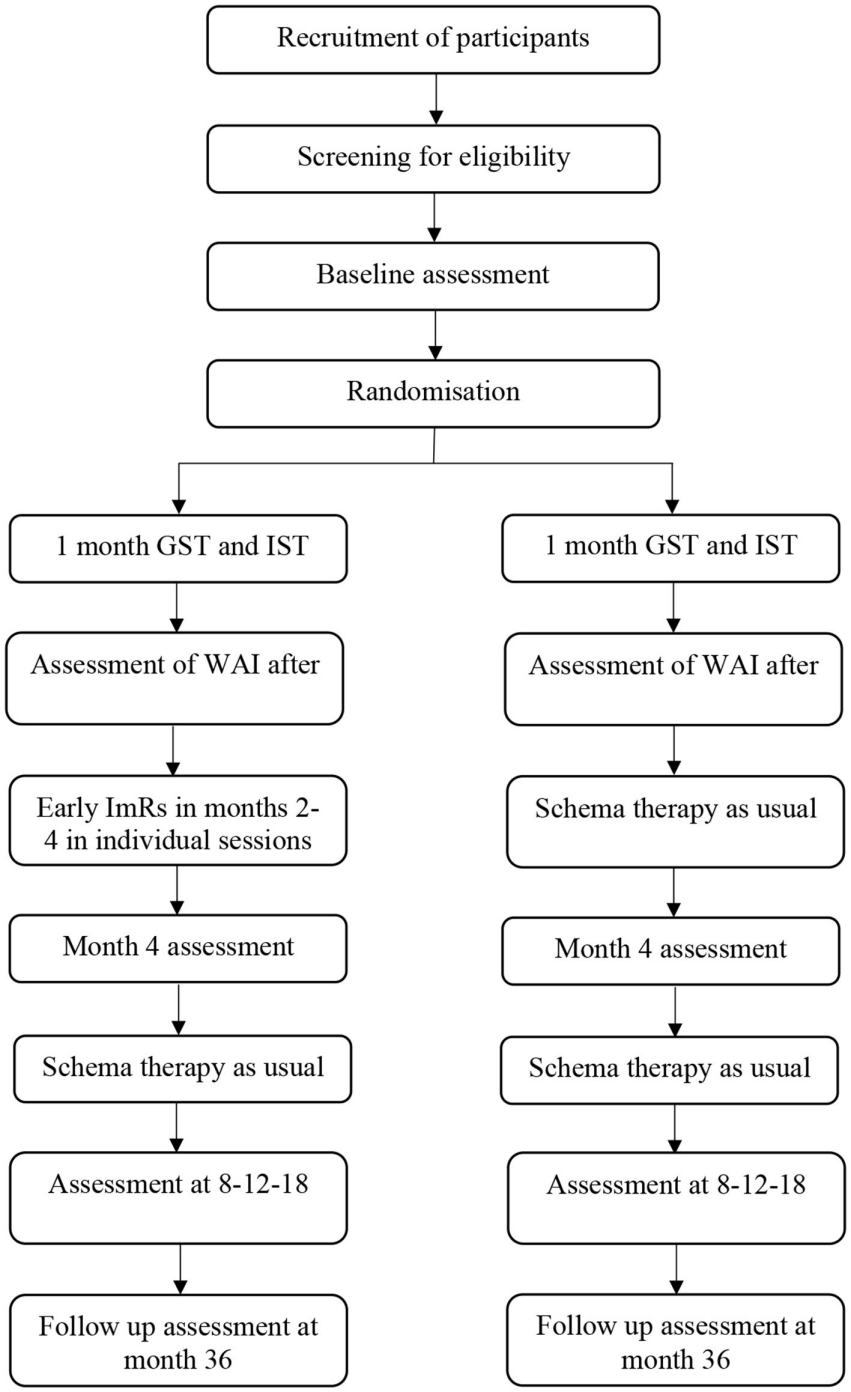
Flowchart of the study design.

Patients from participating mental healthcare centers were asked to participate in the study. At all sites, Dutch-speaking participants were recruited; however, at the Amsterdam site, an additional cohort of English-speaking (non-Dutch) participants was recruited, resulting in one group of patients who received treatment in English. The same standardized measures were used for the Dutch and English patients. Patients with a primary diagnosis of BPD according to DSM-5 were asked to participate in the screening procedure (24). Patients were given both written and verbal information about the study, and after they provided informed consent, the screening process commenced. The sample included BPD patients with and without comorbid PTSD. Figure 1 illustrates the patient flow from recruitment onward; to achieve the intended sample sizes, site coordinators kept the central research team informed of the progress of participant recruitment and any issues that arose during the study.

### Randomization

When participants were eligible for participation and completed baseline assessments, they were randomized to one of the two arms A (early ImRs) or B (STAU) in a 1:1 proportion by an independent researcher, after checking the inclusion and exclusion criteria, using block randomization with randomized block sizes blocks (sizes 4 and 6), stratified by the center (for the Amsterdam site stratified for language) (29, 30). The allocated treatment condition was disclosed to the patient by their therapist during the first treatment session.

### Procedure and assessments

Patients were recruited from participating mental healthcare centers. Patients with a primary diagnosis of BPD, classified using the Structured Clinical Interview for Personality Disorders (SCID-5-PD) (22) by trained clinicians, were asked to participate in the screening process of the study. A check was performed to determine eligibility. This check concerned examining whether participants met the inclusion and exclusion criteria, and their willingness and ability to participate in 2-year individual and group schema therapy.

After the first eligibility check but before randomization, the baseline measurements (see Measures Section) and a motivational/availability interview assessing the participants' motivation were performed by an independent research assistant. After 4, 8, 12, 18, 24, and 36 months, assessments by an independent trained local research assistant blinded to the treatment arm were performed (Table 1). Each assessment took approximately 2.5 h. The results of the assessments were shared with the patients and therapists so that they could be used as routine outcome measures to track and discuss treatment progress. Participants who drop out of treatment and or the study will be asked to continue the study assessments; however, if the participant decides to withdraw from the study, a minimized exit assessment is conducted—if the participant consents, in which the primary outcome measure is assessed. Patients will receive a monetary incentive of 25 euros after completion of all assessments. All group ST sessions are video-recorded, and individual ST sessions are audio-recorded. A random sample will be selected from each site. Independent raters will evaluate a random selection of these recordings to evaluate treatment adherence and competence of group ST with the Group Schema Therapy Treatment Integrity scale (31) and individual ST with Schema Therapist Competency Scale [STCS-I-1; (32)]. The recordings will be destroyed 5 years after the primary study results have been published. The results of the baseline assessments were provided to the therapists to formulate the ST case conceptualization.

**Table 1:** Overview of measures and assessment times.

**Instrument**	**Baseline**	**After 3 sessions**	**4 months**	**8 months**	**12 months**	**18 months**	**24 months**	**36 months**
CTQ-SF	•							
BPDSI	•		•	•	•	•	•	•
WHODAS 2.0	•		•	•	•	•	•	•
WAI-SR		•	•	•	•	•	•	•
BSI	•		•	•	•	•	•	•
PCL-5	•		•	•	•	•	•	•
QoL	•		•	•	•	•	•	•
Happiness	•		•	•	•	•	•	•
SMI-2	•		•	•	•	•	•	•
YSQ-R	•		•	•	•	•	•	•

### Ethical aspects

Patients received verbal and written information about the study, and they were asked to give their written consent after a clear explanation and time (minimum 2 days) for reflection on participation in the study. Participants are allowed to leave the study at any time, keeping their right to alternative treatment at the participating center. Therapists are allowed to withdraw a patient, in consultation with the participant, from treatment only in exceptional situations after consultation with the central investigators. The (research) data collected during this project will be stored and shared in a safe and secure manner using systems that are managed and maintained by the IT departments of the involved institutions and conform to privacy regulations. Due to the sensitivity of our (research) data, especially to protect the privacy of the research participants, as well as participants not having given consent to share their data with others, data on an individual level cannot be shared with others. The study protocol was approved by the local ethics committee of the Department of Psychology at the University of Amsterdam (2019-CP-10845).

### Treatment

ST, developed by Jeffrey Young, derives from cognitive behavioral therapy (CBT) (33). This approach integrates concepts from various theoretical frameworks such as attachment theory, psychodynamic therapy, and experiential therapies. Schema therapy employs extensive experiential techniques in conjunction with cognitive and behavior-focused methods. The therapeutic relationship is defined as “limited reparenting,” where the therapist, within the bounds of a professional therapeutic alliance, adopts a nurturing role akin to a 'supportive parent' for the patient and tries to fulfill the basic needs of the client. The underlying premise of schema therapy is that childhood trauma and unmet fundamental needs, compounded by biological and cultural factors, contribute to the development of early maladaptive schemas, fundamental mental frameworks encompassing self-perception, interpersonal dynamics, and one's outlook on the world. Additionally, the theory posits the existence of dysfunctional schema modes comprising emotional, cognitive, and behavioral states.

The treatment consists of a fixed combination of group sessions and individual sessions. Groups consist of a maximum of nine patients. ST starts with three individual pretreatment sessions over 4 weeks. The main treatment consists of a treatment phase and a maintenance phase. The treatment period lasts a maximum of 18 months and is divided into two phases. The active treatment phase lasts for 12 months and includes weekly group psychotherapy sessions of 90 min, as well as weekly individual psychotherapy sessions with a duration of 45–50 min. This is followed by 6 months of weekly group ST sessions and every 2 weeks individual ST sessions. After 18 months, participants end the group treatment. The maintenance phase lasts for 6 months and consists of biweekly individual ST sessions for the first 3 months, followed by one individual session per month for the next 3 months. In this study, the individual ST has two arms; both commence with 1 month of getting acquainted with the ST concepts, forming a working relationship, and formulating a case conceptualization. In months 2, 3, and 4, patients were allocated to one of two individual schema therapy conditions:

A. Early ImRs: ImRs of early childhood memories during 12 weeks.
B. STAU: Schema therapy as usual. Experiential techniques are allowed but the use of ImRs is not allowed in the first 4 months.

One site delivers intensive day clinical treatment. The treatment duration at this site is 24 months, divided into four phases; phase 1 has a duration of 6 months in which group ST is given three times a week for 90 min plus one individual session with a duration of 45 min. In phase 2, months 7–12: group ST is delivered 2 times a week for 90 min, accompanied by one individual session every 2 weeks, for 45 min. In phase 3, months 13–18, GST is delivered once a week for 90 min, and individual sessions are delivered once every 2 weeks for 3 months and thereafter once a month. In phase 4, one group therapy session is provided every 2 weeks, and individual sessions once every 2 months.

Group ST follows the protocol of Farrell et al. (34) and individual ST the protocol of Arntz and van Genderen (9). Group and individual ST are coordinated during weekly peer supervision meetings. The groups are semi-closed: every 12 sessions, patients can be included until the group is complete, participants who complete 1.5 years of treatment leave the group, and in the following session, new patients enter the group.

#### Use of co-intervention

Patients are allowed to continue medication during the study. However, patients who began taking medication within 3 months before the initial screening were excluded from the study, or they could participate after 3 months. Except for crisis interventions, no other psychological or new pharmacological therapies are permitted during treatment. Medication use is closely monitored throughout the study period.

#### Escape medication/treatment

If the participants experience an acute crisis during the study, they are permitted to use other forms of therapy or medication. The use of such interventions or medication will not result in exclusion from the study but will be recorded, monitored, and reported.

#### Further treatment

After completion of the 1-year follow-up assessment, the therapists who performed individual ST meet the patient for an evaluation, using the outcomes of the assessments; at this point, it is determined whether additional treatment is necessary. The type, frequency, and intensity of the additional treatment are determined by the center's typical indication procedure. If patients request assistance during the 1-year follow-up period, an estimation is performed if the need for assistance outweighs the benefits of the intended 1-year treatment-free period.

#### Dropout, deviation from the treatment protocol, and follow-up

Patients are free to withdraw from the study at any point for any reason. In serious cases, such as when a patient severely disrupts the treatment process for other group members or frequently misses treatment sessions, therapists may remove patients from treatment after consultation with the researchers (pushout). Site coordinators are responsible for finding appropriate alternatives for patients who drop out or are pushed out of treatment. The reasons for the early termination of treatment will be monitored and recorded by the researchers. If patients make significant progress in treatment, therapists may deviate from the treatment length according to the ST protocols and end the treatment earlier or skip sessions. At the end of treatment, therapists advise patients not to start new treatment during the follow-up period and continue applying the techniques they have learned by themselves. Following completion of the follow-up assessment, the former therapist discusses the results with the patient and helps them determine whether additional therapy is necessary. If a patient requires further therapy, the therapist will assist them in identifying the type of therapy needed and provide a referral if the therapy is not available at the treatment site.

#### Therapists, training, and supervision

Therapists must meet the following criteria: (1) At least junior level training and registration according to the Dutch ST register; (2) (if group schema therapist) completed a recognized course in GST according to the Farrell and Shaw ST model (34); and (3) completed a 2-day ImRs training in which they learn the ImRs protocol as described in the study by Arntz and van Genderen (9) and Arntz (35). The therapists will meet each other for 1 h every week for peer supervision and may consult the study's ST supervisors (AK and AA) if necessary.

### Measures

#### Diagnostic and baseline assessments

The Dutch DSM-5 versions of the Structured Clinical Interview for DSM-5 Disorders—SCID-5-S and SCID-5-P—were used to assess syndromal and personality disorders during screening (22, 36). A checklist is used to assess the inclusion and exclusion criteria. Furthermore, at baseline, the Childhood Trauma Questionnaire-Short Form [CTQ-SF; (37)], PCL-5, Young Schema Questionnaire-Short Form, Schema Mode Inventory, and (after preparatory session 3 with the individual therapist) WAI self-report questionnaire (38) were assessed.

Adverse childhood experiences are assessed by the Childhood Trauma Questionnaire-Short Form [CTQ-SF; (37)], a self-reported questionnaire comprising 25 items. These items are rated on a 5-point Likert scale, ranging from 1 (never true) to 5 (very often true). The CTQ-SF encompasses the following five subcategories: sexual abuse, physical abuse, emotional abuse, physical neglect, and emotional neglect. The CTQ-SF has demonstrated satisfactory validity and reliability, with Cronbach's alpha ranging from 0.53 (physical neglect) to 0.91 (sexual abuse) (39, 40). To assess the severity of trauma exposure, norm scores are available, categorizing individuals into four groups: none, low, moderate, and severe (41).

#### Primary outcome measures

The primary focus of the study is to determine changes in the severity and frequency of DSM-5 BPD manifestations, assessed with the Borderline Personality Disorder Severity Index version 5 (BPDSI-5) (42). The BPDSI-5 is a structured clinical interview that employs 70 items to assess the severity of BPD within the preceding 3 months. The items assess different manifestations of the nine BPD traits outlined in the DSM-5. Each of these nine traits is evaluated using multiple items. The frequency of occurrence indicated by each item is evaluated using an 11-point Likert scale, ranging from 0 (absent) to 10 (daily). However, the 'identity disturbance' trait diverges, utilizing a 5-point Likert scale from 0 (not present) to 4 (dominant), which is then multiplied by 2.5. For each BPD trait, a subscale score is calculated consisting of the mean of the pertinent items (range 0–10). The total score of the BPDSI-5 score ranges from 0 to 90, reflecting overall severity. The psychometric properties of the BPDSI-5 are good, with a high level of agreement between different raters and high internal consistency. The scale has a cutoff point of 14.93 with scores above, indicating clinical levels of BPD dysfunction and exhibiting considerable specificity (1.00) and sensitivity (0.97). Additionally, Giesen-Bloo et al. (42) concluded that a reliable positive change is indicated by an improvement of at least 11.70 points.

#### Secondary outcome measures

1. Treatment dropout. The number of sessions and the date on which treatment dropout (or pushout) occurred will be monitored.

2. The working alliance self-report (WAI-SR) is used to measure patients' thoughts and feelings about the working relationship with the individual ST therapist (38). The WAI-SR has three scales that measure different aspects of the therapeutic alliance. each aspect is measured by four items. The scales are (1) agreement on the tasks of therapy, (2) agreement on the goals of therapy, and (3) the development of an affective bond. the items are scored on a 5-point likert scale. the goal, task, and bond domains have scores ranging from 5 to 20. Higher scores indicate a better therapeutic alliance. the WAI-SR demonstrates strong internal consistency; values of cronbach's alpha for each of the three subscales range from 0.81 to 0.90, while the total score has cronbach's alpha of 0.91. the WAI-SR also exhibits considerable reliability, with a test–retest reliability of 0.93 (95% CI 0.83–0.97) (43). in terms of its construct validity, the WAI-SR exhibits high correlations with other measures of the therapeutic alliance, such as a correlation of 0.80 with the california psychotherapy alliance scale and a correlation of 0.74 with the helping alliance questionnaire (44). The first assessment of the WAI-SR took place 3 weeks after starting therapy, during the preparation phase, because some experience with the treatment and contact with the therapist is needed to answer the WAI questions. note that this is before the imrs sessions start in the experimental arm A. participants were instructed to fill out the WAI after this session and return it before the fourth individual session in a sealed envelope to the secretary (and not give it to the therapist) to prevent their ratings from being influenced by therapists reading their ratings. the later WAIs are part of the computerized battery that participants fill in during regular assessments, which are assessed by an independent research assistant.

3. The WHO disability assessment schedule 2.0 36-item interview version (WHODAS 2.0) (45) is used to assess general functioning, measuring impairment in six domains: cognition, mobility, self-care, getting along, life activities, and participation. In this study, the WHODAS is administered by the independent research assistant. All items are assessed using a 5-point likert scale, with scores ranging from 1 (non) to 5 (extreme or cannot do), based on the person's experience in the past 30 days. a summary score will be derived using the complex scoring method (45). The WHODAS 2.0 has excellent psychometric properties. Test–retest studies of the 36-item scale in countries across the world found it to be highly reliable, with an intraclass coefficient of 0.69–0.89 at the item level, 0.93– 0.96 at the domain level, and 0.98 at the overall level. cronbach's alpha levels were generally very high (0.94–0.96 for domains and 0.98 for total score) (45).

4. Early maladaptive schemas were assessed using the Dutch Young Schema Questionnaire-3 Short Form [YSQ-3SF; (46)]. In the current study, the sum score of the schemas from the disconnection/rejection domain is used to test the hypothesis that early ImRs leads to a faster reduction of schemas from this domain. The sum of the scores of the other domains is used for exploratory purposes. The YSQ-3SF consists of 90 items with a 6-point Likert scale ranging from 1 (completely untrue of me) to 6 (describes me perfectly) for measuring 18 schemas, as formulated by Young et al. (33). The psychometric properties of the YSQ-3SF are good, with a test–retest reliability of 0.98 and adequate internal consistency for all 18 scales (α > 0.70). In addition, a significant association has been found between the schemas measured by this questionnaire and features of several personality disorders (46, 47).

5. The severity of posttraumatic stress symptoms is measured using the Post-Traumatic Checklist for DSM-5 (PCL-5) (48). The PCL-5 is a self-report measure for PTSD symptoms with 20 items which are rated on a 5-point Likert scale, from 0 (not at all) to 4 (extremely), assessing the 20 DSM-5 symptoms of PTSD. The PCL-5 provides a severity score between 0 and 80, and the developers suggest that a PCL-5 cutoff score between 31 and 33 is indicative of probable PTSD. Although a diagnosis cannot be made on the basis of the PCL-5 alone, clinicians can look at individual items to determine whether the number of symptoms required by the DSM-5 is present. The PCL-5 has good psychometric properties with good internal consistency (α = 0.96), test–retest reliability (*r* = 0.84), and convergent and discriminant validity (49–51).

6. Quality of Life will be assessed with the EuroQol EQ-5D-5L (52, 53). Five dimensions (mobility, self-care, usual activities, pain/discomfort, and anxiety/depression) are measured and categorized into five severity levels: no problems, slight problems, moderate problems, severe problems, and extreme problems. There are 3,125 possible health states defined by combining one level from each dimension, ranging from 11,111 (full health) to 55,555 (worst health). EQ-5D-5L health states are converted into a single index ‘utility' score using a scoring algorithm based on (Dutch) public preferences. The EQ-5D-5L has good psychometric properties. The intraclass correlation of the EQ-5D-5L ranges from 0.73 to 0.84 for the summary index, and the instrument has good construct validity (54).

7. The SMI is a self-report instrument that consists of 143 items on 16 schema modes that are scored on six-point Likert scales (1 = *completely untrue*, 6 = *completely true)*. It measures the extent to which dysfunctional (vulnerable child, angry child, enraged child, impulsive child, undisciplined child compliant surrender, detached protector, self-soother, self-aggrandizer, bully and attack, punitive parent, demanding parent, healthy adult, perfectionistic overcontroller, and suspicious overcontroller) and functional schema modes (happy child and healthy adult) were present at the time of assessment (55). It is an adaptation of the original SMI containing 270 items (56) and a short SMI containing 118 items (57). Its subscales have satisfactory to high internal consistency (Cronbach's α ranges from 0.79 to 0.96) (57).

8. General psychopathological symptoms as an index of the severity of syndromal disorders are assessed with the Brief Symptom Inventory [BSI; (58)], a short version of the Symptom-Check-List (SCL-90-R). The BSI is a self-reporting instrument that measures general psychiatric symptoms. It measures nine symptom dimensions: somatization, obsession-compulsion, interpersonal sensitivity, depression, anxiety, hostility, phobic anxiety, paranoid ideation, and psychoticism with 53 items scored on a 5-point Likert scale, ranging from 0 (not at all) to 4 (extremely). The BSI has good reliability and validity. The internal consistency of the total score is 0.96, and the test–retest reliability is 0.90 (59). Normative data are available for both clinical and non-clinical samples of adolescents (over 13 years) and adults (60).

9. Happiness is assessed with the 1-item happiness question validated in more than 30 countries (61). General happiness, in the weeks prior to the assessment, is assessed by a single 7-point Likert scale item ranging from 1 (completely unhappy) to 7 (completely happy). The reliability and validity of the happiness item are good to excellent, with a test–retest reliability of 0.87 (62).

10. Medication and mental healthcare use are monitored during each assessment.

### Study assessment moments

Outcome instruments are assessed at baseline (0 months), at 4 months (12 sessions of ImRs or ST-as-Usual), after 8 months of treatment, after 1 year of treatment, at the end of group therapy (after 18 months of treatment), and after 24 months (after the end of individual treatment). One year later, a 3-year follow-up assessment is performed. Table 1 presents an overview of the measurements and the timeframe of the assessments.

The WAI, assessing the therapeutic alliance with the individual therapist as experienced by the participant, cannot be assessed at baseline because the patient and therapist have not yet met each other. Therefore, the first WAI assessment will take place after the third individual session, that is, before the ImRs in the experimental arm is started.

### Statistical analysis

All analyses are planned to be carried out using SPSS or R statistical software. Dimensional outcomes will be analyzed with multilevel analysis using all available data (intent-to-treat principle). Depending on the empirically found distribution of the dependent variable, the appropriate (generalized) linear mixed model will be chosen (e.g., in the case of non-normal distribution, gamma, negative binomial, or Tweedie distributions with a loglink). Time models might be linear, curvilinear, or piecewise, depending on which model fits the data best. The fixed part will contain the main effects of time and treatment arm and their interaction. The random part will contain a covariance structure for the repeated part with the best fit to the data (e.g., AR1, ARMA11, and compound symmetry) and a random intercept for group and/or participant and/or random slopes for time. Adding a random part for each group is important because of the interdependence of assessments of participants per GST group. If necessary, that is in case of substantial differences between treatment arms at baseline, differences at baseline will be controlled for by adding covariates to the model. Multiple imputation methods will be used to handle missing values at the item level. However, completely missing measurements will not be imputed as this is not necessary for multilevel analysis (63). Dropout will be analyzed using survival analysis, with the treatment arm as a factor. If data allow, a multilevel survival analysis will be used to take the dependency of observation by group into account (group as random intercept). The study will use (G)LMM analyses to test predictors and moderators, including working alliances, schema modes, and PTSD symptoms. The analysis will involve adding predictor, predictor x time, predictor x treatment, and predictor x time x treatment interactions to the model.

### Qualitative data analysis

With regard to the qualitative study among therapists and patients, we plan to employ a thematic analytic approach, combining both a top-down approach with pre-formulated themes and a bottom-up approach that allows new themes to emerge from the interviews (64). Inter-rater agreement will be assessed using blinded double coding. Rather than aiming for statistical representativeness, our sample prioritizes diversity. Participants will be selected based on a range of factors: treatment site, sex, age, ethnic background, treatment outcomes (success or failure), and treatment completion or premature termination (65).

### Data management, storage, monitoring, and dissemination

Data are collected using a web-based tool called Lotus, developed by the University of Amsterdam. The tool is used to monitor the progress of patients and remind research assistants and junior researchers of upcoming assessments. The patients are registered in Lotus and assigned a unique identifier to maintain their anonymity. The personal information of patients is stored in a secure location and is accessible only to authorized personnel. The data are collected through a secure online survey software called Qualtrics (66) and stored on a secure server accessible only to authorized researchers. The Study Board, consisting of the steering committee, shares ownership of the data and makes the final decisions. The data will not be publicly available because of their sensitive nature. The results of this study will be disseminated through publications in scientific journals and presentations at conferences. Training and supervision will also be offered to participating clinicians to facilitate implementation. The site coordination committee is responsible for addressing the potential difficulties in the execution of the study.

### Safety

Previous studies (see Background Section) suggest that there is no significant risk for patients undergoing the treatments being investigated. To minimize any potential harm, patients with acute suicidality are excluded from the study. In cases of serious adverse events (SAEs), which include events resulting in death, hospitalization, or significant disability, the junior researcher will be notified within 24 h by the local site coordinators. The relatedness of the SAE to the intervention will be evaluated by the PIs and reported to the ethical committee of the University of Amsterdam. Because the occurrence of SAEs is expected to be low, no specific hypotheses were formulated with regard to conditions and SAEs. However, the number and type of SAEs will be reported per study arm in the outcome paper.

## Discussion

The current trial provides an opportunity to investigate the effect of early trauma treatment on the course of BPD severity during ST. When early trauma treatment by ImRs leads to a faster decrease in BPD manifestations, ST for BPD patients might be further shortened, which in turn leads to a reduction in direct and indirect healthcare costs and waitlists for ST. Currently, there is a great need for efficient and effective treatments for BPD as a considerable number of patients with BPD do not receive evidence-based treatment for BPD, and waitlists for specialized BPD treatments are very long [e.g., for the Dutch situation: (67)].

The current study also provides insight into the working mechanisms of ST, which will contribute to the identification of key elements of ST. A better understanding of the mechanisms of change provides an opportunity to further improve ST for BPD. Furthermore, studying these mechanisms can improve our understanding of BPD and the variables associated with the course of BPD. Studying the underlying mechanisms will also help to discover important mechanisms of change that cut across different types of therapy, contributing to a better understanding of psychological interventions in general (68).

The LUCY trial has several strengths. First, it has good external validity. The study is conducted in regular outpatient healthcare centers among a representative group of patients which will contribute to the generalizability of the findings to routine clinical care.

Second, it has a good methodological quality. In this study, a multimethod assessment approach is used (i.e., self-report and semi-structured interviews, as well as a qualitative study next to the quantitative study), protocolized treatments are used, an assessment of treatment integrity will be performed, and long-term effects are investigated by means of a follow-up at 3 years. The semi-structured interviews are administered by independent, trained research assistants who are blinded to the treatment arm to prevent interviewer bias.

Third, this trial focuses on changes in a broad spectrum of comorbid and associated symptoms of BPD (e.g., general functioning, PTSD symptoms, general psychopathological complaints, quality of life, and happiness), by which it can be examined whether early trauma treatment in ST is effective for a wide range of symptoms and levels of functioning.

Fourth, the study tests the hypothesis that early application of ImRs leads to reduced treatment retention, a prediction that follows from the original ST protocol. In other words, the focus is not only on possible positive outcomes but also on the possible negative effects of early trauma processing.

Fifth, the focus is not on the treatment of PTSD in participants with a double diagnosis of BPD and PTSD but on treating any kind of negative early childhood experiences that contributed to the development of BPD. The conceptualization of this study and the hypotheses being investigated are based on an acknowledgment of the importance of such adverse events in the etiology of personality pathology and in the treatment of BPD. This is important because sadly, many adverse childhood experiences are often neglected even when there is a diagnosis of PTSD (69).

The following limitations of this study should be noted. First, in this RCT, early trauma treatment in ST is not compared to early trauma treatment in other evidence-based therapies for BPD, such as MBT, DBT, and TFP, nor is there a waitlist control group. When patients improve in both treatment arms and no differences between arms are found, it cannot be ruled out that non-treatment-specific factors (e.g., time and positive effects due to attention) can explain the improvement. This RCT has two arms, because a third treatment arm would make the execution of the trial much more complex because of the necessity of the availability of a third (evidence-based) treatment at each site, and a much higher N would be needed. In the current trial, ST-as-Usual is the control condition and the primary research question focuses on comparing the differences between the two ST conditions. Second, the study was powered only to detect medium effect sizes. Small effects of early trauma treatment on BPD severity and/or comorbidities and associated symptoms of BPD may not be detected. Third, the results cannot be generalized to treatments other than ST and ImRs. Fourth, the analyses that we will perform do not control for the total amount of ImRs that has been applied. This study will only investigate the effect of early application of ImRs vs. the current standard ST protocol, which does not prescribe the number of times ImRs is applied. With a positive effect of early ImRs, an interesting question for follow-up studies would be what the effects are of early ImRs, when the number of ImRs sessions is the same in both conditions.

In conclusion, this study is the first to compare the effectiveness of early trauma treatment in ST with regard to the course of BPD, treatment dropout, working alliance, and other secondary variables. Given the high prevalence of adverse childhood experiences in BPD, such insights are needed to further improve therapies for BPD. Additionally, the underlying mechanisms and predictors of (differential) treatment outcomes will be investigated, providing more insight into how treatments work and for whom treatments work. Therefore, this study will significantly extend our knowledge of trauma treatment for BPD.

## Ethics statement

This study protocol is approved by the Local Ethical Committee of the Department of Psychology of the University of Amsterdam (2019-CP-10845). Written informed consent will be obtained from the participants.

## Author contributions

AK and NB wrote the first draft of the manuscript and were involved in the implementation and daily coordination of the study. AA served as the principal investigator, who wrote the initial concept and design of this trial. All authors contributed to the study design and have read, commented on, and approved the manuscript.

## References

[1] Arntz A. Imagery rescripting as a therapeutic technique: review of clinical trials, basic studies, research agenda. J Exp Psychopathol. (2012) 3:189–208. 10.5127/jep.024211

[2] Kip A Schoppe L Arntz A Morina N. Efficacy of imagery rescripting in treating mental disorders associated with aversive memories - an updated meta-analysis. J Anxiety Disord. (2023) 10:2772. 10.1016/j.janxdis.2023.102772 37699277

[3] Morina N Lancee J Arntz A. Imagery rescripting as a clinical intervention for aversive memories: a meta-analysis. J Behav Ther Exp Psychiatry. (2017) 55:6–15. 10.1016/j.jbtep.2016.11.003 27855298

[4] Raabe S Ehring T Marquenie L Arntz A Kindt M. Imagery Rescripting as a stand-alone treatment for posttraumatic stress disorder related to childhood abuse: a randomized controlled trial. J Behav Ther Exp Psychiatry. (2022) 77:101769. 10.1016/j.jbtep.2022.101769 36113906

[5] Boterhoven de Haan KL Lee CW Fassbinder E Voncken MJ Meewisse M van Es SM . Imagery rescripting and eye movement desensitisation and reprocessing for treatment of adults with childhood trauma-related post-traumatic stress disorder: IREM study design. BMC Psychiat. (2017) 17:1–12. 10.1186/s12888-017-1330-2 28472933 PMC5418842

[6] Bohus M Dyer AS Priebe K Kruger A Kleindienst N Schmahl C . Dialectical behaviour therapy for post-traumatic stress disorder after childhood sexual abuse in patients with and without borderline personality disorder: a randomized controlled trial. Psychother Psychosom. (2013) 82:221–33. 10.1159/000348451 23712109

[7] Harned MS Korslund KE Linehan MM. A pilot randomized controlled trial of Dialectical Behaviour Therapy with and without the Dialectical Behaviour Therapy prolonged Exposure protocol for suidicidal and self-injuring women with borderline personality disorder and PTSD. Behav Res Ther. (2014) 55:7–17. 10.1016/j.brat.2014.01.008 24562087 PMC3987949

[8] Rüfenacht EN Shaverin L Bateman A Fonagy P Taubner S . Theorie und Praxis der Trauma-fokussierten Mentalisierungsbasierten Therapie. Psychotherapie. (2023) 68:458–65. 10.1007/s00278-023-00686-2

[9] Arntz A van Genderen H. Schema therapy for borderline personality disorder. Chichester: John Wiley & Sons. (2011). 10.1002/9781119962830.ch30

[10] Lobbestael J Arntz A Bernstein DP. Disentangling the relationship between different types of childhood maltreatment and personality disorders. J Pers Disord. (2010) 24:285–95. 10.1521/pedi.2010.24.3.285 20545495

[11] Bozzatello P Rocca P Baldassarri L Bosia M Bellino S. The role of trauma in early onset borderline personality disorder: a biopsychosocial perspective. Front Psychiatry. (2021) 12:721361. 10.3389/fpsyt.2021.721361 34630181 PMC8495240

[12] Porter C Palmier-Claus J Branisky A Mansell W Warwick H Varese F. Childhood adversity and borderline personality disorder: a meta-analysis. Acta Psychiatrica Scandinavica. (2020) 141:6–20. 10.1111/acps.13118 31630389

[13] Jowett S Karatzia T Albert I. Multiple and interpersonal trauma are risk factors for both post-traumatic stress disorder and borderline personality disorder: A systematic review on the traumatic backgrounds and clinical characteristics of comorbid post-traumatic stress disorder/borderline personality disorder groups versus single-disorder groups. Psychology and Psychother. (2020) 93:621–38. 10.1111/papt.12248 31444863

[14] Hernandez A Arntz A Gaviria AM Labad A Gutiérrez-Zotes JA. Relationships between childhood maltreatment, parenting style and borderline personality disorder criteria. J Pers Disord. (2012) 26:727–36. 10.1521/pedi.2012.26.5.727 23013341

[15] Pilkington PD Bishop A Younan R. Adverse childhood experiences and early maladaptive schemas in adulthood: a systematic review and meta-analysis. Clini Psychol Psychother. (2021) 28:569–84. 10.1002/cpp.2533 33270299

[16] Carr SN Francis AJ. Early maladaptive schemas and personality disorder symptoms: An examination in a non-clinical sample. Psychol Psychother. (2010) 83:333–49. 10.1348/147608309X481351 25268482

[17] Slotema CW Blom JD Niemantsverdriet MBA Deen M Sommer IEC. Comorbid diagnosis of psychotic disorders in borderline personality disorder: prevalence and influence on outcome. Front Psychiatry. (2018) 9:84. 10.3389/fpsyt.2018.00084 29593589 PMC5861147

[18] Hafkemeijer L de Jongh A van der Palen J Starrenburg A. Eye movement desensitization and reprocessing (EMDR) in patients with a personality disorder. Eur J Psychotraumatol. (2020) 11:1838777. 10.1080/20008198.2020.1838777 33425243 PMC7755323

[19] Arntz A Mensink K Cox WR Verhoef RE van Emmerik AA Rameckers SA . Dropout from psychological treatment for borderline personality disorder: a multilevel survival meta-analysis. Psychol Med. (2023) 53:668–86. 10.1017/S0033291722003634 36453183 PMC9975988

[20] Bosch M Arntz A. *Imagery rescripting for patients with posttraumatic stress* disorder: a qualitative study of patients' and therapists' perspectives about the elements of change. Cogn Behav Pract. (2021) 30:18–34. 10.1016/j.cbpra.2021.08.001

[21] Wibbelink CJM Arntz A Grasman PPP Sinnaeve R Boog M Bremer OMC . Towards optimal treatment selection for borderline personality disorder patients (BOOTS): a study protocol for a multicenter randomized clinical trial comparing schematherapy and dialectical behavior therapy. BMC Psychiatry. (2022) 22:89. 10.1186/s12888-021-03670-9 35123450 PMC8817780

[22] Arntz A Kamphuis JH Derks J. SCID-5-P. Gestructureerd klinisch interview voor DSM-5 Persoonlijkheidsstoornissen. Amsterdam: Boom. (2017).

[23] Arntz A van den Hoorn M Cornelis J Verheul R van den Bosch WM de Bie AJ. Reliability validity of the borderline personality disorder severity index. J Pers Disord. (2003) 17:45–59. 10.1521/pedi.17.1.45.24053 12659546

[24] American Psychiatric Association. Diagnostic and Statistical Manual of Mental Disorders. (5th edition) (DSM-5). Washington DC: Author. (2013).

[25] Twisk JWR. Sample size calculations. In: Analysis of Data from Randomized Controlled Trials. Cham: Springer. (2021).

[26] Arntz A Jacob GA Lee CW Brand-de Wilde OM Fassbinder E Harper RP . Effectiveness of predominantly group schema therapy and combined individual and group schema therapy for borderline personality disorder: a randomized clinical trial. JAMA Psychiat. (2022) 79:287–99. 10.1001/jamapsychiatry.2022.0010 35234828 PMC8892362

[27] Moerbeek M van Breukelen GJP Berger MPF. Optimal Designs for Multilevel Studies In:de Leeuw E Meijer J, editors. Handbook of Multilevel Analysis. New York, NY: Springer. (2008), 177–206.

[28] Sealed Envelope Ltd. Power Calculator for Binary Outcome Superiority Trial. (2012). Available online at: https://www.sealedenvelope.com/power/binary-superiority/ (accessed April 10, 2023).

[29] Efird J. Blocked randomization with randomly selected block sizes. Int J Environ Res Public Health. (2011) 8:15–20. 10.3390/ijerph8010015 21318011 PMC3037057

[30] Pocock SJ. Clinical Trials: A Practical Approach. New York, NY: Wiley. (1983).

[31] Baljé A. Treatment Integrity Check of Group Schema Therapy for Avoidant Personality Disorder. Leiden: University of Leiden: Internal Document. (2018).

[32] Young JE Fosse G. Schema Therapist Competency Scale (STCS-I-1). (2008). Available online at: https://www.isstonline.com (accessed April 10, 2023).

[33] Young JE Klosko JS Weishaar ME. Schema Therapy. New York: Guilford. (2003) 254:653–8.

[34] Farrell JM Reiss N Shaw IA. The Schema Therapy Clinician's Guide: A Complete Resource for Building and Delivering Individual, Group and Integrated Schema Mode Treatment Programs. John Wiley & Sons (2014).

[35] Arntz A. Imagery rescripting for personality disorders: healing early maladaptive schemas. In:Thoma NC McKay D, editors. Working with Emotion in Cognitive Behavioral Therapy: Techniques for Clinical Practice. New York: Guilford Press. (2015) p. 175–202.

[36] Arntz A Kamphuis JH Derks JL. SCID-5-S: Gestructureerd klinisch interview voor DSM-5 Syndroomstoornissen: Nederlandse vertaling van Structured Clinical Interview for DSM-5 Disorders-Clinician Version (SCID-5-CV) en User's Guide for the Structured Clinical Interview for DSM-5 Disorders-Clinician Version en delen van Structured Clinical Interview for DSM-5 Disorders-Research Version (SCID-5-RV). (2018).

[37] Bernstein DP Stein JA Newcomb MD Walker E Pogge D Ahluvalia T . Development and validation of a brief screening version of the Childhood Trauma Questionnaire. Child Abuse Negl. (2003) 27:169–90. 10.1016/S0145-2134(02)00541-0 12615092

[38] Horvath AO Greenberg LS. Development validation of the Working Alliance Inventory. J Couns Psychol. (1989) 36:223–33. 10.1037/0022-0167.36.2.223

[39] Karos K Niederstrasser N Abidi L Bernstein DP Bader K. Factor structure, reliability, and known groups validity of the German version of the Childhood Trauma Questionnaire (Short-form) in Swiss patients and nonpatients. J Child Sex Abus. (2014) 23:418–30. 10.1080/10538712.2014.896840 24641795

[40] Thombs BD Bernstein DP Lobbestael J Arntz A. A validation study of the Dutch Childhood Trauma Questionnaire-Short Form: factor structure, reliability, known-groups validity. Child abuse and neglect. (2009) 33:518–23. 10.1016/j.chiabu.2009.03.001 19758699

[41] Bernstein D Fink L. Childhood Trauma Questionnaire: A Retrospective Self-Report. San Antonio, TX: The Psychological Corporation (1998).

[42] Giesen-Bloo JH Wachters LM Schouten E Arntz A. The Borderline Personality Disorder Severity Index-IV: Psychometric evaluation and dimensional structure. Pers Individ Dif. (2010) 49:136–41. 10.1016/j.paid.2010.03.023

[43] Munder T Wilmers F Leonhart R Linster HW Barth J. Working Alliance Inventory-Short Revised (WAI-SR): psychometric properties in outpatients and inpatients. Clini Psychol. (2010) 17:231–9. 10.1002/cpp.658 20013760

[44] Hanson WE Curry KT Bandalos DL. Reliability generalization of working alliance inventory scale scores. Educ Psychol Meas. (2002) 62:659–73. 10.1177/0013164402062004008

[45] Ustün TB Chatterji S Kostanjsek N Rehm J Kennedy C Epping-Jordan J . Developing the World Health Organization disability assessment schedule 2.0. Bull World Health Organ. (2010) 88:815–23. 10.2471/BLT.09.067231 21076562 PMC2971503

[46] Rijkeboer MM Videler AC Rossi G van Alphen SPJ Legra M. Nederlandse vertaling van de Young Schema Questionnaire - Short Form Version 3 (YSQ-3S). (2020). Available online at: https://www.~schematherapie.nl/Deskundigen/Vragenlijsten (accessed April 10, 2023).

[47] Bach B Simonsen E Christoffersen P Kriston L. The Young Schema Questionnaire 3 Short Form (YSQ-S3). Eur J Psychol Assessm. (2017) 33:134–43 10.1027/1015-5759/a000272

[48] Weathers FW Litz BT Keane TM Palmieri PA Marx BP Schnurr PP. The PTSD Checklist for DSM-V (PCL-5). In: The National Center for PTSD. (2013). Available online at: www.ptsd.va.gov.

[49] Blevins CA Weathers FW Davis MT Witte TK Domino JL. The posttraumatic stress disorder checklist for DSM-5 (PCL-5): development and initial psychometric evaluation. J Trauma Stress. (2015) 28:489–98. 10.1002/jts.22059 26606250

[50] Bovin MJ Marx BP Weathers FW Gallagher MW Rodriguez P Schnurr PP . Psychometric properties of the PTSD Checklist for Diagnostic and Statistical Manual of Mental Disorders-Fifth Edition (PCL-5) in veterans. Psychol Assessm. (2016) 28:1379–91. 10.1037/pas0000254 26653052

[51] Wortmann JH Jordan AH Weathers FW Resick PA Dondanville KA Hall-Clark B . Psychometric analysis of the PTSD Checklist-5 (PCL-5) among treatment-seeking military service members. Psychol Assess. (2016) 28:1392. 10.1037/pas0000260 26751087

[52] Herdman M Gudex C Lloyd A Janssen MF Kind P Parkin D . Development and preliminary testing of the new five-level version of EQ-5D (EQ-5D-5L). Qual Life Res. (2011) 20:1727–36. 10.1007/s11136-011-9903-x 21479777 PMC3220807

[53] Rabin R Charro FD. EQ-SD: a measure of health status from the EuroQol Group. Ann Med. (2001) 33:337–43. 10.3109/07853890109002087 11491192

[54] Janssen MF Pickard AS Golicki D Gudex C Niewada M Scalone L . Measurement properties of the EQ-5D-5L compared to the EQ-5D-3L across eight patient groups: a multi-country study. Qual Life Res. (2013) 22:1717–27. 10.1007/s11136-012-0322-4 23184421 PMC3764313

[55] Lobbestael J Van Vreeswijk MF Arntz A. An empirical test of schema mode conceptualizations in personality disorders. Behav Res Ther. (2008) 46:854–60. 10.1016/j.brat.2008.03.006 18460405

[56] Young JE Arntz A Atkinson T Lobbestael J Weishaar ME van Vreeswijk MF . The Schema Mode Inventory. New York: Schema Therapy Institute. (2007).

[57] Lobbestael J van Vreeswijk MF Spinhoven P Schouten EG Arntz A. Reliability and validity of the short Schema Mode Inventory (SMI). Behav Cognit Psychother. (2010) 4:437–58. 10.1017/S1352465810000226 20487590

[58] Derogatis LR Melisaratos N. The brief symptom inventory: an introductory report. Psychol Med. (1983) 13:595–605. 10.1017/S0033291700048017 6622612

[59] De Beurs E Zitman FG. The Brief Symptom Inventory (BSI): Reliability and validity of a practical alternative to SCL-90. Maandblad Geestelijke Volksgezondheid. (2006) 61:120–41.

[60] Derogatis LR. The Brief Symptom Inventory (BSI): Administration, Scoring and Procedures Manual. Minneapolis, MN: National Computer Systems (1993).

[61] Veenhoven R. (2011). Available online at: http://worlddatabaseofhappiness.eur.nl/hap_quer/hqs_fp.htm (accessed April 10, 2023).

[62] Abdel-Khalek AM. Measuring happiness with a single-item scale. Social Behav Personal. (2006) 34:139–50. 10.2224/sbp.2006.34.2.139

[63] Twisk J de Boer M de Vente W Heymans M. Multiple imputation of missing values was not necessary before performing a longitudinal mixed-model analysis. J Clini Epidemiol. (2013) 66:1022–8. 10.1016/j.jclinepi.2013.03.017 23790725

[64] Braun V Clarke V. Using thematic analysis in psychology. Qual Res Psychol. (2006) 3:77–101. 10.1191/1478088706qp063oa

[65] Schreier M. Sampling generalization. In: The Sage Handbook of Qualitative Data Collection. Sage (2018). p. 84–98.

[66] Qualtrics (2005). Available online at: https://www.qualtrics.com (accessed April 10, 2023).

[67] NZA (2019). Available online at: https://puc.overheid.nl/nza/doc/PUC_297810_22/1/ (accessed April 10, 2023).

[68] Moldovan R. Mechanisms of change in psychotherapy: methodological and statistical considerations. Cogn Brain, Behav. (2015) 19:4.

[69] Rameckers SA van Emmerik AA Bachrach N Lee CW Morina N Arntz A. The impact of childhood maltreatment on the severity of childhood-related posttraumatic stress disorder in adults. Child Abuse Neglect. (2021) 120:105208. 10.1016/j.chiabu.2021.105208 34332332

